# Investigation of Rho-Kinase Expressions and Polymorphisms in Mantle Cell Lymphoma Patients

**DOI:** 10.4274/tjh.2015.0193

**Published:** 2016-05-16

**Authors:** Didar Yanardağ Açık, Mehmet Yılmaz, İbrahim Sarı, Serdar Öztuzcu, Zeynel A. Sayıner, Salih Subari, Abdullah T. Demiryürek

**Affiliations:** 1 Gaziantep University Faculty of Medicine, Department of Internal Medicine, Division of Hematology, Gaziantep, Turkey; 2 Gaziantep University Faculty of Medicine, Department of Pathology, Gaziantep, Turkey; 3 Gaziantep University Faculty of Medicine, Department of Medical Biology, Gaziantep, Turkey; 4 Gaziantep University Faculty of Medicine, Department of Internal Medicine, Gaziantep, Turkey; 5 Gaziantep University Faculty of Medicine, Department of Medical Pharmacology, Gaziantep, Turkey

**Keywords:** Lymphoma, Expression, polymorphism, Rho-kinase

## Abstract

**Objective:**

Mantle cell lymphoma (MCL) is a rare but aggressive form of B-cell non-Hodgkin lymphoma characterized by excessive expression of cyclin D1. Intracellular signaling enzyme Rho-kinase (ROCK) can contribute to cellular migration, proliferation, and differentiation, as well as tumor development and metastasis. However, ROCK gene and protein expressions or polymorphisms have never been investigated in MCL patients. The purpose of this study was to investigate the role of ROCK gene and protein expressions in MCL patients. We also examined ROCK2 gene polymorphisms in this study.

**Materials and Methods:**

A total of 60 patients with MCL and 60 healthy controls were included in this retrospective study. Hematoxylin and eosin-stained lymph node tissue slides in the entire archive were reevaluated and used for immunohistochemistry, gene expression, and polymerase chain reaction studies.

**Results:**

In immunohistochemical studies, there were significant increases in ROCK1 (p=0.0009) and ROCK2 (p<0.0001) protein expressions in MCL patients when compared with the control group. Although a marked increase in ROCK1 gene expression (p=0.0215) was noted, no significant change was observed in ROCK2 gene expression in MCL patients. Seven ROCK2 polymorphisms were studied, but the results showed no significant differences between the groups.

**Conclusion:**

This is the first study to show that ROCK1 gene and ROCK protein expressions may contribute to the development of MCL.

## Abstract (tr)

**Amaç:**

Mantle hücreli lenfoma (MHL) siklin D1’in aşırı ekspresyonuyla karakterize B-hücreli Hodgkin dışı lenfomanın nadir fakat agresif bir şeklidir. İntraselüler sinyal enzimi olan Rho-kinaz (ROCK), hücre migrasyonu, proliferasyonu, farklılaşması yanında tümör gelişimi ve metastazına da katkıda bulunur. Fakat MHL hastalarında ROCK gen ve protein ekspresyonları veya polimorfizmleri araştırılmamıştır. Bu çalışmanın amacı, MHL hastalarında ROCK gen ve protein ekspresyonlarının rolünü araştırmaktı. Biz bu çalışmada ROCK2 gen polimorfizmleri de araştırdık.

**Gereç ve Yöntemler:**

Bu retrospektif çalışmaya 60 MHL hastası ve 60 sağlıklı kontrol dahil edildi. Bütün arşivde hematoksilin ve eosin boyalı lenf düğümü kesitleri yeniden incelendi ve immünohistokimya, gen ekspresyonu ve polimeraz zincir reaksiyonu çalışmaları için kullanıldı.

**Bulgular:**

İmmünohistokimyasal çalışmada, kontrol grubuyla karşılaştırıldığında MHL hastalarında ROCK1 (p=0,0009) ve ROCK2 protein ekspresyonlarında (p<0,0001) anlamlı artış vardı. MHL hastalarında ROCK1 gen ekspresyonunda (p=0,0215) anlamlı artış bulunmasına karşın ROCK2 gen ekspresyonunda anlamlı değişiklik gözlenmedi. Yedi ROCK2 polimorfizmi çalışıldı, fakat sonuçlar gruplar arasında anlamlı farklılıklar göstermedi.

**Sonuç:**

Bu çalışma, ROCK1 gen ve ROCK protein ekspresyonlarının MHL gelişimine katkısı olabileceğini gösteren ilk çalışmadır.

## INTRODUCTION

Mantle cell lymphoma (MCL) is an aggressive B-cell-type non-Hodgkin lymphoma characterized by cyclin D1 overexpression and occurs more commonly in advanced ages and in males [1,2]. MCL is a rare subtype accounting for about 6% of all non-Hodgkin lymphoma cases. During the development of MCL, via t(11;14) (q13;q32) translocation, the BCL1 gene (11q13) and immunoglobulin heavy chain gene (14q32) come together, and hence BCL1 is upregulated. As a consequence of this translocation, excessive synthesis of cyclin D1 protein promotes the cell cycle progression (to S-/G2-phase) via cyclin-dependent kinase 4 and 6 activation in an uncontrolled manner. A minority (about 5%) of cases are cyclin D1-negative, and these cases often exhibit high expression of cyclin D2 or D3 [3]. Phenotypically, MCL is positive for the B-cell markers CD5, CD20, and CD79a. In MCL cells, CD10 and CD23 are usually negative. There is also overexpression of SOX-11 in the nuclei in most cases [4]. Overexpression of SOX-11 has been described as a diagnostic marker for MCL, with the absence of SOX-11 a characteristic of indolent MCL [5].

No single genetic lesion that can give rise to MCL has been identified. Molecular studies including single nucleotide polymorphisms (SNPs) have revealed a large number of chromosomal alterations in MCL [6,7,8,9]. Several copy number aberrations have been found to be correlated with genomic complexity in MCL cases [10].

Most patients are diagnosed at an advanced stage, and extranodal sites are often involved [11]. Even though patients with MCL often respond to therapy, the responses are usually partial and most patients eventually relapse [12]. There is currently no proven curative therapy and no standard of care has been established for initial or subsequent lines of therapy. Therefore, ideal treatment regimens for MCL are still being investigated and studies indicate that intracellular signaling pathways may be important targets in the treatment of MCL.

Rho-kinase (ROCK) signaling has been implicated in various cellular functions downstream of Rho GTPases. Rho GTPases are important regulators of cancer cell proliferation, survival, invasion, and metastasis. More recently, crucial functions of Rho GTPases in the regulation of tumor stroma, including endothelial cells, immune cells, and cancer-associated fibroblasts, as well as in the formation of microvesicles, have been reported [13]. ROCK is a serine-threonine protein kinase with multiple downstream effects. Two isoforms of ROCK protein, ROCK1 and ROCK2, have been characterized. The ROCK isoforms are encoded by separate genes on human chromosomes 18q11 (ROCK1) and 2p24 (ROCK2) [14,15]. ROCK is substantially involved in a wide range of fundamental cellular functions, such as proliferation, differentiation, adhesion, contraction, metabolism, and apoptosis. ROCK signaling enhances myosin-mediated contractility and drives amoeboid migration, which is associated with certain types of carcinoma, lymphomas, and leukemia [15,16,17]. Increased expression of the ROCK proteins promotes tumor cell proliferation and contributes to the metastatic behavior of some cancers [15]. Several of the ROCK substrates are prominent players in the development of cancer and its associated phenotypes. For example, the tumor suppressor phosphatase and tensin homolog (PTEN), which is frequently inactivated in melanoma, as well as c-Jun N-terminal Kinase (JNK)-interacting protein-3, an inhibitor of JNK signaling that is upregulated in melanoma, are inhibited by ROCK phosphorylation [17]. It has been shown that the sustained activation of ROCK is sufficient to induce cell cycle progression and increase cyclin D1 expression in NIH 3T3 fibroblasts [18]. Furthermore, ROCK activation also increases the expression of cyclin D1 in vascular smooth muscle cells [19]. In this study, the contribution of both ROCK isoforms in MCL was investigated. We also explored the possible role of the ROCK gene and protein expressions in MCL and tested the hypothesis that genetic variations in the ROCK2 gene may increase the risk of MCL.

## MATERIALS AND METHODS

### Patients

In the present study, tissue samples of 60 patients diagnosed with MCL between 2006 and 2012, and those of 60 healthy adults who underwent lymph node biopsy for any reason but were not diagnosed with any malignant disease and were reported to have only hyperplasia by the pathology department, were investigated retrospectively. The study was approved by the local ethics committee.

Clinical and laboratory information at the date of first diagnosis was recorded and overall survival was calculated as time from diagnosis to death or to the date when the patient was seen for the last time. Patients were identified from the pathological records and all cases were confirmed by histological evaluation. All demographic and clinical characteristics as well as prognostic factors of the study cases were collected from files. The prognosis of patients was based on the Mantle Cell Lymphoma International Prognostic Index (MIPI), which is calculated on the basis of four independent prognostic factors (age, performance status, serum lactate dehydrogenase level, and leukocyte count).

### Immunohistochemistry

Formalin-fixed, paraffin wax-embedded blocks from each case were selected for immunohistochemical studies using the antibodies against ROCK1 and ROCK2. Hematoxylin and eosin-stained lymph node tissue slides were used for immunohistochemistry. Control tissue sections were made from the lymph node biopsies of the healthy subjects. Sections of 4 µm were cut from paraffin-embedded tissue blocks onto silane-coated slides. Sections were heated to 60 °C for 20 min prior to deparaffinization with xylene solution. Sections were then stained using the Bond Polymer Refine Detection Kit (Bond #DS9800) in an automated slide processing system (Bond-Max, Leica Microsystems, Buffalo Grove, IL, USA). ROCK1 (rabbit monoclonal, EP786Y, ab45171, Abcam, Cambridge, UK) and ROCK2 (rabbit polyclonal, ab71598, Abcam, Cambridge, UK) were used for ROCK1 and ROCK2 immunostaining, respectively. The percentage of cells staining was evaluated and intensity (–, +, ++, or +++) was scored from 0 to 3 [20].

### DNA Isolation and Genotyping

DNA isolation was done with the paraffin blocks using the QIAamp DNA FFPE Tissue Kit (Cat. No. 56404). Obtained DNA was measured with a UV spectrophotometer (Epoch Biotek, Winooski, VT, USA) and prepared for the study. Various SNPs in the gene region coding ROCK2 were investigated. Criteria for the choice of SNPs used were: 1) relatively high minor allele frequencies in Caucasians; 2) location within the exonic and intronic sites that could potentially impact ROCK expression and function; and 3) suitability for the Fluidigm dynamic array chip designing, i.e. with no high G/C levels. Reference numbers of SNPs for the ROCK2 gene were rs2290156 in intron 30, rs965665 in intron 3, rs10178332 in intron 3, rs2230774 (Thr431Asn) in exon 10, rs2230774 (Thr431Ser) in exon 10, rs6755196 in intron 1, and rs726843 in intron 13. Polymorphisms were analyzed in genomic DNA using the 96.96 Dynamic Array on the BioMark HD system (Fluidigm, South San Francisco, CA, USA). Digital PCR Analysis software (Fluidigm, South San Francisco, CA, USA) was used to process the data after the reaction [21].

### Gene Expression

Ribonucleic acid (RNA) was extracted from formalin-fixed, paraffin wax-embedded blocks using the High Pure RNA Isolation Kit (Cat. No. 03 270 289 001, Roche Diagnostics, Mannheim, Germany) as described by the manufacturer. The obtained RNA was prepared for the study by being measured with UV spectrophotometry. cDNA synthesis was performed with the Transcriptor First Strand cDNA Synthesis Kit (Roche Diagnostics, Mannheim, Germany) according to manufacturer’s protocol. Gene expression analysis was then done using a BioMark HD device (Fluidigm, South San Francisco, CA, USA) that utilizes a fluorescent PCR method. Data were analyzed using the 2-ΔCt method according to the following formula: ΔCt=CtROCK-CtGAPDH, where Ct=threshold cycle [22].

### Statistical Analysis

Data were expressed as mean ± standard deviation (SD) or percentage unless otherwise indicated. Statistical analysis was performed using GraphPad InStat version 3.05 (GraphPad Software Inc., San Diego, CA, USA). For comparisons of the differences between mean values of two groups, the unpaired Student t-test was used. The chi-square test for independence and Fisher exact tests were used for calculation of the significance of differences in genotype and allele frequencies. The Pearson test was used to identify the correlations. The Mann-Whitney U test was used to detect significant differences between immunohistochemical scores and compare the gene expression data between groups. All statistical tests and p-values were two-sided, and p<0.05 was considered statistically significant.

## RESULTS

**Figure 1:**
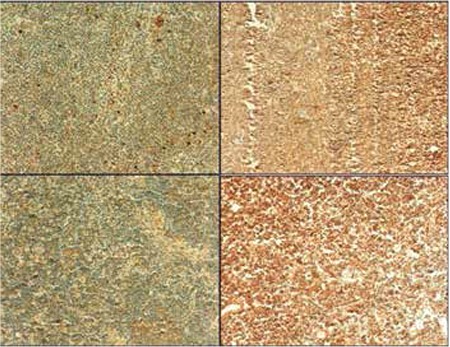
Histopathologic images of ROCK staining. Immunohistochemical staining for lymph node tissues with ROCK1 in control (a) and in mantle cell lymphoma patients (b), and ROCK2 staining in control (c) and in mantle cell lymphoma patients (d). Original magnification 200x.

**Figure 2:**
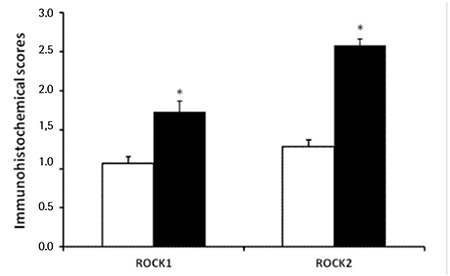
Comparison of the immunohistochemical scores for lymph node ROCK1 and ROCK2 staining in healthy controls (n=60, white bars) and in patients with mantle cell lymphoma (n=60, black bars). Values are given as mean ± SEM. *p=0.0009 and p<0.0001 values were obtained for ROCK1 and ROCK2, respectively.

**Figure 3:**
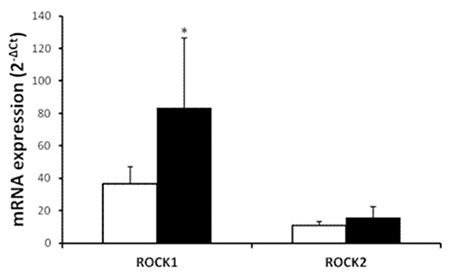
Comparison of the lymph node ROCK1 and ROCK2 gene messenger ribonucleic acid expressions in healthy controls (white bars, n=41) and in patients with mantle cell lymphoma (black bars, n=44). Values are given as mean ± SEM. *p=0.0215 and p=0.9194 values were obtained for ROCK1 and ROCK2 gene, respectively.

Demographic and clinical characteristics of MCL patients and controls are outlined in Table 1. There were no statistically significant differences between patients and control groups in terms of sex and age distribution. Immunohistochemical study of the lymph node tissues revealed that ROCK1 and ROCK2 staining was more marked in the patient group (Figure 1). A widespread stronger positivity for ROCK1 and ROCK2 staining was observed in the cytoplasm of the lymph node cells from MCL patients. The ROCK distribution displayed a similar pattern between control and MCL sections. There were marked increases in ROCK1 (1.72±1.08, p=0.0009) and ROCK2 (2.58±0.62, p<0.0001) staining scores in the lymph nodes of the patient group when compared to controls (1.07±0.66 for ROCK1 and 1.28±0.69 for ROCK2; Figure 2). Correlations between the prognostic factors and ROCK in MCL patients are shown in Table 2. It was found that there were significant negative correlations between number of drug therapies and ROCK1 and ROCK2 protein expressions. However, positive correlation was found between age and ROCK1 expression. We also noted a positive correlation between ROCK1 and ROCK2 expressions in MCL patients (Table 2). No significant differences were found between MCL patients and the control group in terms of 7 ROCK2 gene polymorphisms (Table 3). There was a marked increase in ROCK1 gene expression in the patient group when compared to controls (p=0.0215). However, no significant change was observed in ROCK2 gene expression (p=0.9194; Figure 3).

## DISCUSSION

This study provides the first evidence that ROCK1 and ROCK2 protein expressions and ROCK1 gene expression were increased in MCL patients. However, no marked change in ROCK2 gene expression was observed. There were also no significant associations between ROCK2 gene polymorphisms and MCL cases.

Information regarding underlying biology and pathogenesis constantly increases, forming the basis of molecularly targeted treatment approaches in MCL [23]. Increased protein expressions of two ROCK isoforms have been found to be associated with different types of cancer [15,24]. In the present study, elevation of the ROCK1 protein expression in MCL patients may be due to increase in the ROCK1 gene expression. However, we found an increase in ROCK2 protein, but not gene, expression in MCL patients, suggesting that other mechanisms are involved in the ROCK2 protein expression. The underlying mechanism of this observation is currently unknown, and it may require further evaluation with other techniques. Lane et al. [25] investigated the expressions of ROCK1 and ROCK2 in human breast cancer and showed that expression of ROCK1, at both messenger RNA (mRNA) and protein levels, is much higher in human breast tumor tissue compared with normal tissue. Conversely, ROCK2 levels do not seem to vary significantly between normal and tumor tissue, although a significant decrease was seen in ROCK2 mRNA levels in patients who died from breast cancer [25]. ROCK1 is also highly expressed in tumor tissues from osteosarcoma patients [26]. High expression of ROCK2 protein has been found to be associated with more aggressive behavior in hepatocellular carcinomas [27]. Elevated ROCK2 protein expression levels have also been reported in colon and bladder cancers and are associated with shorter disease-free survival in patients with bladder cancer [28,29]. Collectively, these data may indicate that ROCK is a potential therapeutic target in MCL.

It is known that reactive oxygen species (ROS) can directly act on the Rho/ROCK signaling pathway [30]. The RhoA/ROCK pathway may also modulate ROS generation. ROCK is documented to stimulate expression of NADPH oxidase and consequent generation of ROS [31]. Continued oxidative stress can lead to chronic inflammation, which in turn could mediate cancer [32]. It has been shown that application of the specific ROCK inhibitors produces suppression of tumor formation, growth, and metastasis [33,34,35], while specific activation of ROCK signaling has been shown to lead to increased tumor cell dissemination and angiogenesis [36]. It was also reported that ROCK inhibitors inhibited the growth of cancer cells and their invasion, and increased their sensitivity to chemotherapeutics [34,37,38]. Taken together, these findings imply that ROCK inhibitors may be beneficial in targeted cancer treatment.

We have observed a marked positive correlation of ROCK1 protein expression with age of the patients. However, no correlation was found between ROCK1 and ROCK2 protein expressions between overall and disease-free survival. These data may imply that ROCK has no marked effect on survival in these patients. In addition, there were significant negative correlations between ROCK1 and ROCK2 expressions and number of drug therapies in the present study. The underlying reason for this negative correlation is not known, but these findings may suggest that short duration of intensive chemotherapy may lead to increased ROCK1 and ROCK2 expressions.

There are only limited numbers of published studies related to ROCK polymorphisms in humans. A recent study demonstrated that ROCK2 gene polymorphisms are significantly associated with colorectal cancer [39] or metastases of breast cancer [40]. However, we have found no support for a role of the studied variants in the ROCK2 gene in risk of MCL in the present study. This may be due to the differences in pathogenesis between different types of cancer as well as the small number of cases in the present study.

## CONCLUSION

In summary, our data strongly suggest that ROCK expressions may contribute to the development of MCL. This study provides novel insights into mechanisms of lymphomagenesis. Our findings may provide an important insight into the future development or use of potential therapeutic approaches, such as ROCK inhibitors, for patients with MCL. The results of the present study may also imply that upregulation of ROCK may represent a prognostic factor in MCL, and ROCK may be a potential target for MCL diagnosis and therapy. Further studies are also required to verify these findings in a larger cohort.

## Ethics

Ethics Committee Approval: The study was approved by the local ethics committee, Informed Consent: It was taken.

## Figures and Tables

**Table 1:**
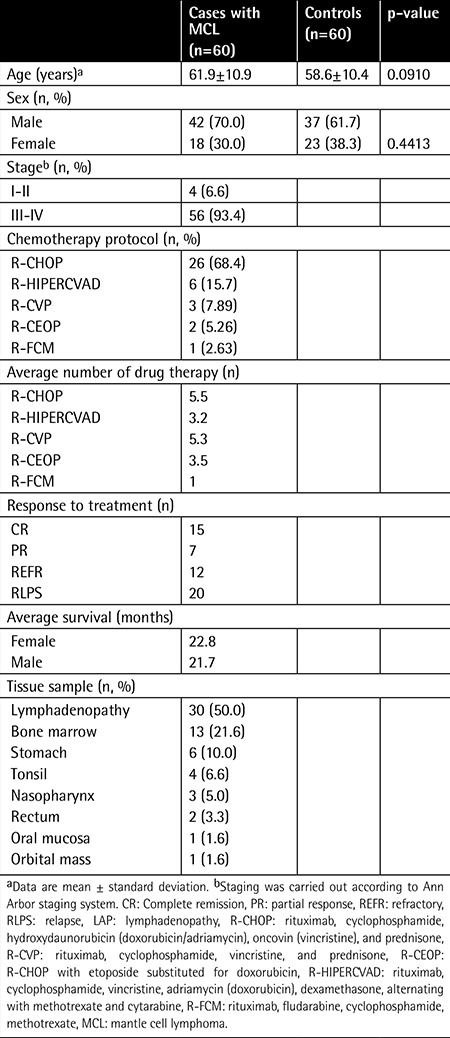
Demographic and clinical characteristics of the study cases.

**Table 2:**
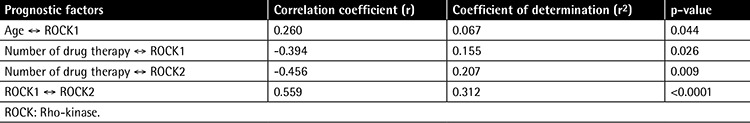
Significant correlations between the prognostic factors and Rho-kinase protein expressions in mantle cell lymphoma patients.

**Table 3:**
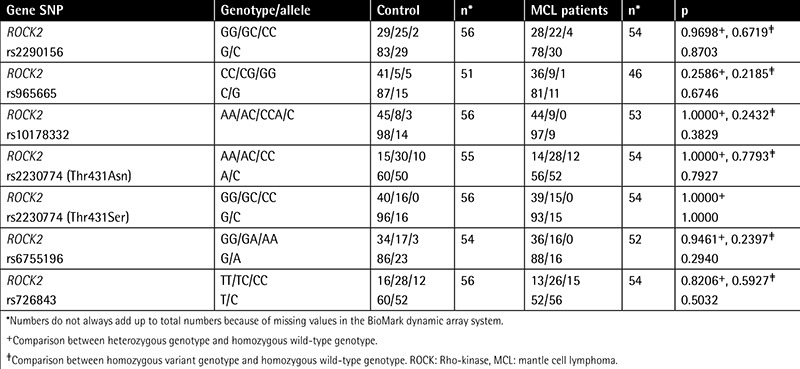
Genotype and allele distributions of ROCK2 gene polymorphisms in patients and control groups.
